# Duplication of the internal maxillary artery: Anatomical and clinical considerations

**DOI:** 10.1177/15910199221142094

**Published:** 2022-11-27

**Authors:** Irene E Harmsen, Cha-ney Kim, Eef J Hendriks, Antti Lindgren, Timo Krings

**Affiliations:** 1 Division of Interventional Neuroradiology, Department of Diagnostic Radiology, 26625 Toronto Western Hospital, University of Toronto, Toronto, ON, Canada; 2 Division of Neuroradiology, Department of Diagnostic Radiology, University of British Columbia, Vancouver, BC, Canada; 3 Department of Clinical Radiology, 60650 Kuopio University Hospital, Kuopio, Finland; 4 School of Medicine, Faculty of Health Sciences Institute of Clinical Medicine, 220881 University of Eastern Finland, Kuopio, Finland

**Keywords:** Internal maxillary artery, duplication, fenestration, cerebral angiography, anatomic variation

## Abstract

Duplication of the internal maxillary artery (IMAX) results from a failed regression of either the embryological superficial or deep ring and is reported to be exceedingly rare. We present a patient with this rare anatomical variant who was treated by endovascular technique in the clinical context of an acute oropharyngeal hemorrhage.

## Introduction

Cerebral vessel fenestration or duplication represents a rare anatomical variation often found incidentally, without clinical relevance.^1,2^ Basilar artery fenestrations, more aptly named unfused basilar artery segments, may be associated with aneurysms given their structural resemblance to a branching point, whereas their association with arteriovenous malformations or developmental venous anomalies is likely coincidental.^3^ Fenestration within the intracranial arterial system occurs most frequently at the vertebral, basilar, and middle cerebral arteries.^3,4^ It is extremely rare to have a fenestration of the internal maxillary artery (IMAX), and this variation has only been described in a few cases in the literature.

An artery's so-called fenestrated appearance can result from a number of different embryological remodeling or pathological processes, including duplication, non-fusion, true fenestration, or dissection. Duplication refers to the presence of two patent embryological vessels resulting from the failure of the usual regression of one of the vessels—these can be seen at the extradural vertebral artery or the anterior communicating artery complex. Non-fusion occurs when two paired embryological vessels fail to fuse during development, resulting in distinct patent channels—the most common one being along the basilar artery. True fenestration is very rare and results from a vessel being pierced by a nerve or other vessel. Finally, in a dissection, the intimal tear along the wall of the artery may reopen into the parent vessel, thus generating two separate channels on angiographic studies. This article describes a patient with a duplication of the IMAX.

## Case report

Written informed consent was obtained from the patient presented herein. A 63-year-old male presented with acute oropharyngeal hemorrhage with a clinical history of left tonsillar squamous cell carcinoma treated 17 years prior with surgery and chemoradiation. He had been recently started on rivaroxaban for an unprovoked segmental pulmonary embolism. A multiphase computed tomography (CT) angiogram of the head and neck vasculature was performed, which showed an arterial extravasation with belated pooling of contrast in the region of the right medial pterygoid muscle, which was identified as the source of the patient's oropharyngeal hemorrhage. Since the bleeding site could not be identified on direct visualization, oropharyngeal packing was performed. The patient was subsequently intubated and transferred to our institution for endovascular treatment. There was a decrease in hemoglobin of 40 mg/dl over 24 h but with no further hemorrhage. He underwent digital subtraction angiography using a Philips Allura biplane angiography system (Eindhoven, the Netherlands) with selective angiograms performed on the external carotid artery (ECA). This included 3D rotational angiography with multiplanar reconstructions, including maximum intensity projections (MIPs) and superselective angiograms of the IMAX and the ascending pharyngeal artery. Endovascular coil embolization of the distal oropharyngeal portion of the right ascending pharyngeal artery was performed, given the location of arterial blush on the prior CT angiogram.

**Figure 1.**
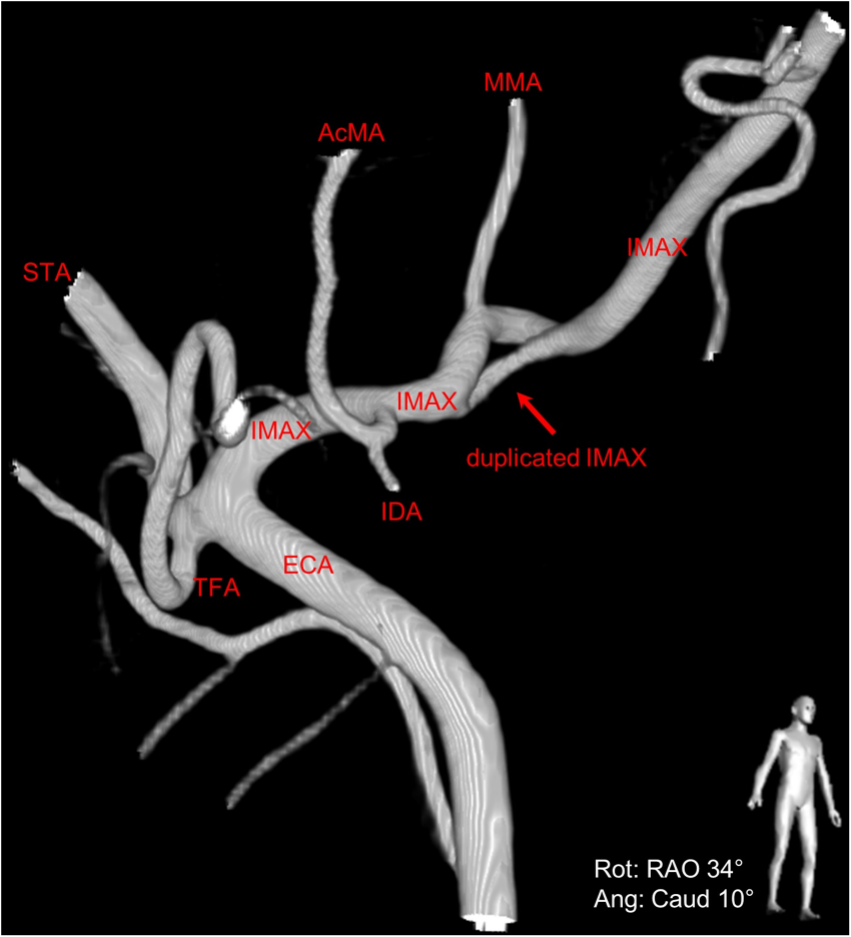
Right anterior oblique view of 3D digital subtraction angiography of the duplicated right internal maxillary artery (IMAX) (red arrow).

AcMA, accessory meningeal artery; ECA, external carotid artery; IDA, inferior dental artery; MMA, middle meningeal artery; STA, superficial temporal artery; TFA, transverse facial artery.

**Figure 2.**
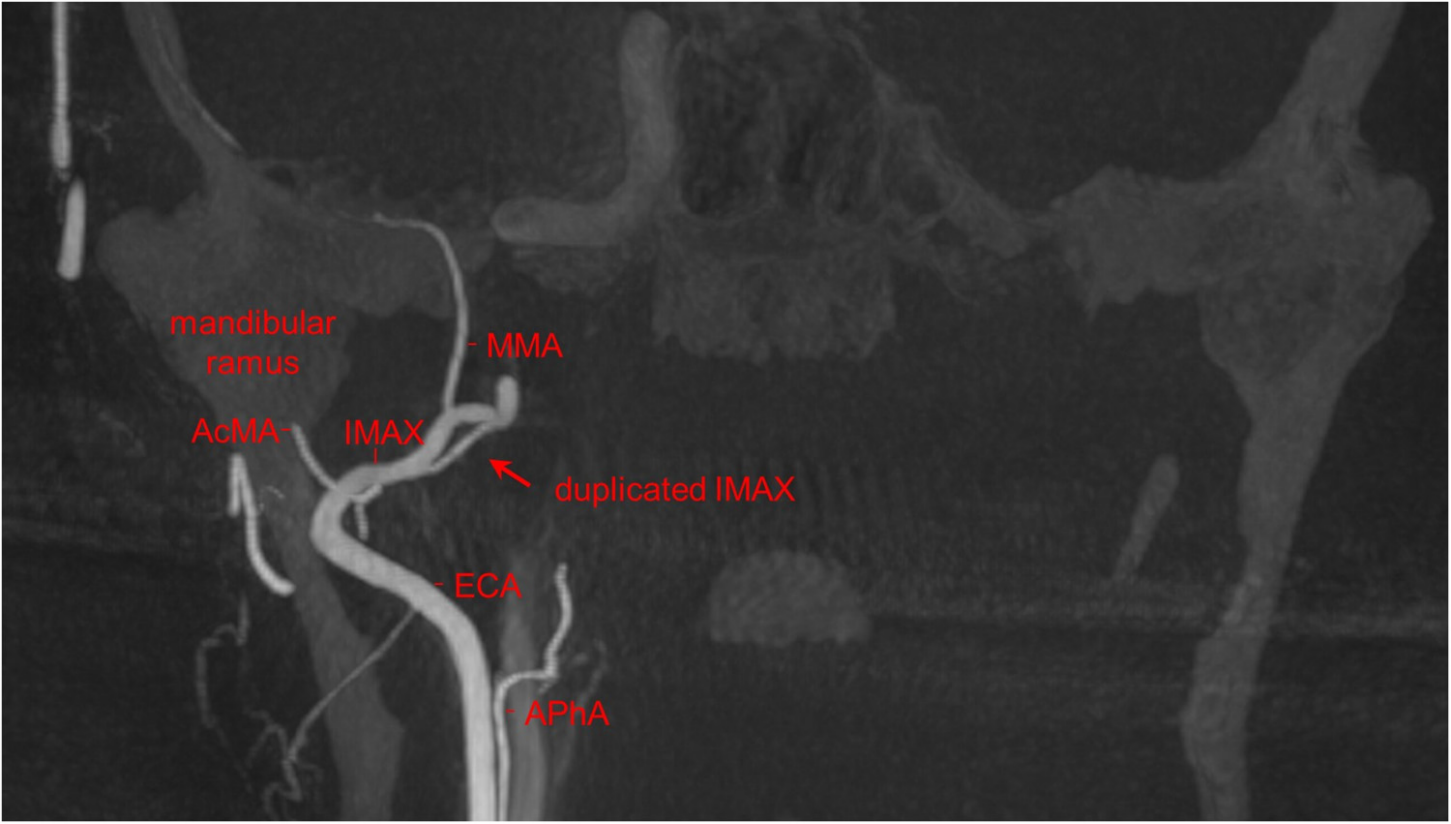
Coronal maximum intensity projection (MIP) from the 3D rotational angiography from a right ECA injection. The duplicated right internal maxillary (IMAX) artery (red arrow) is shown in relation to the right mandible.

AcMA, accessory meningeal artery; APhA, ascending pharyngeal artery; ECA, external carotid artery; MMA, middle meningeal artery.

**Figure 3.**
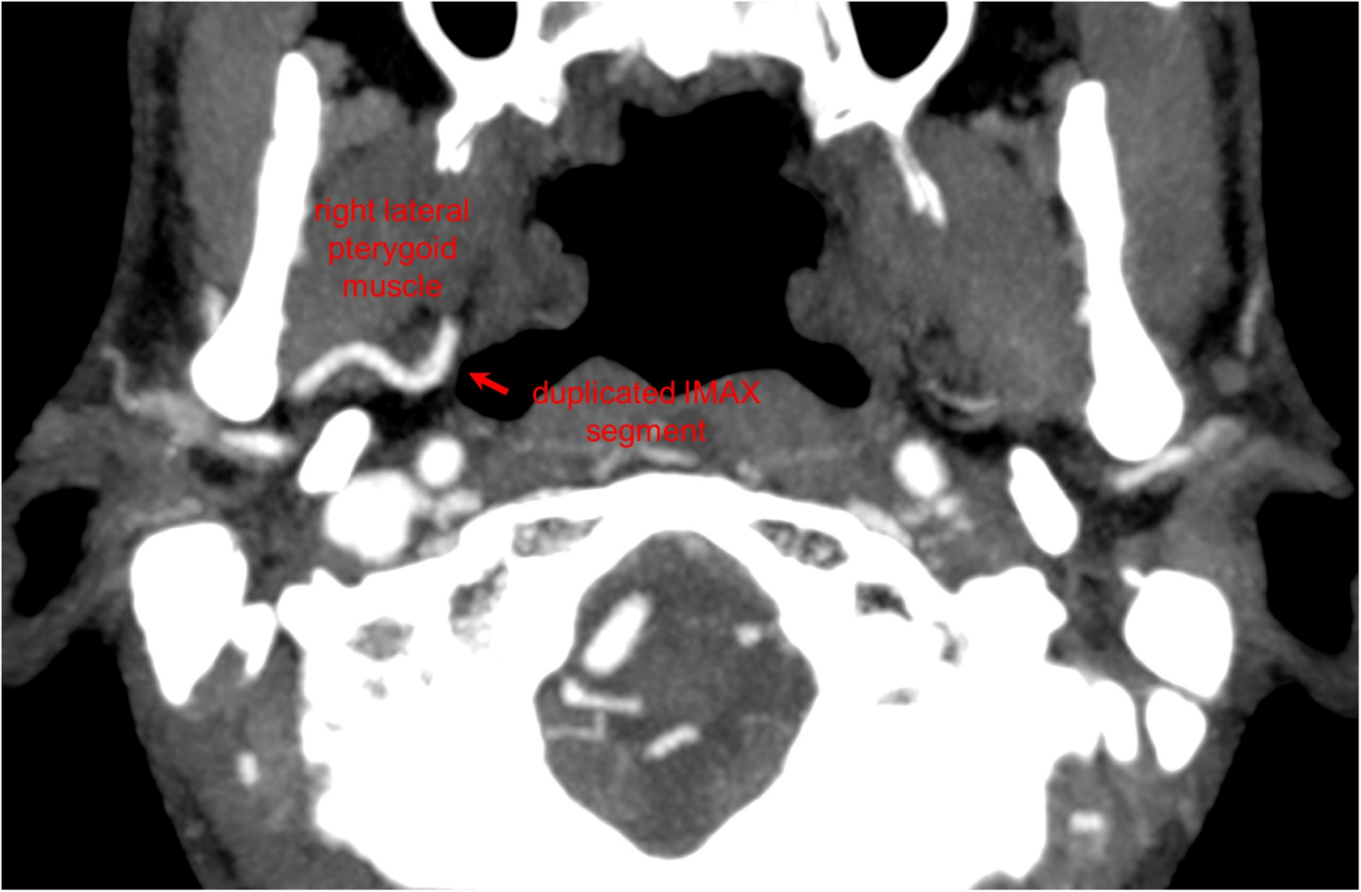
Axial maximum intensity projection (MIP) CT image demonstrates a deep course of the duplicated right internal maxillary artery (IMAX) segment relative to the right lateral pterygoid muscle.

**Figure 4.**
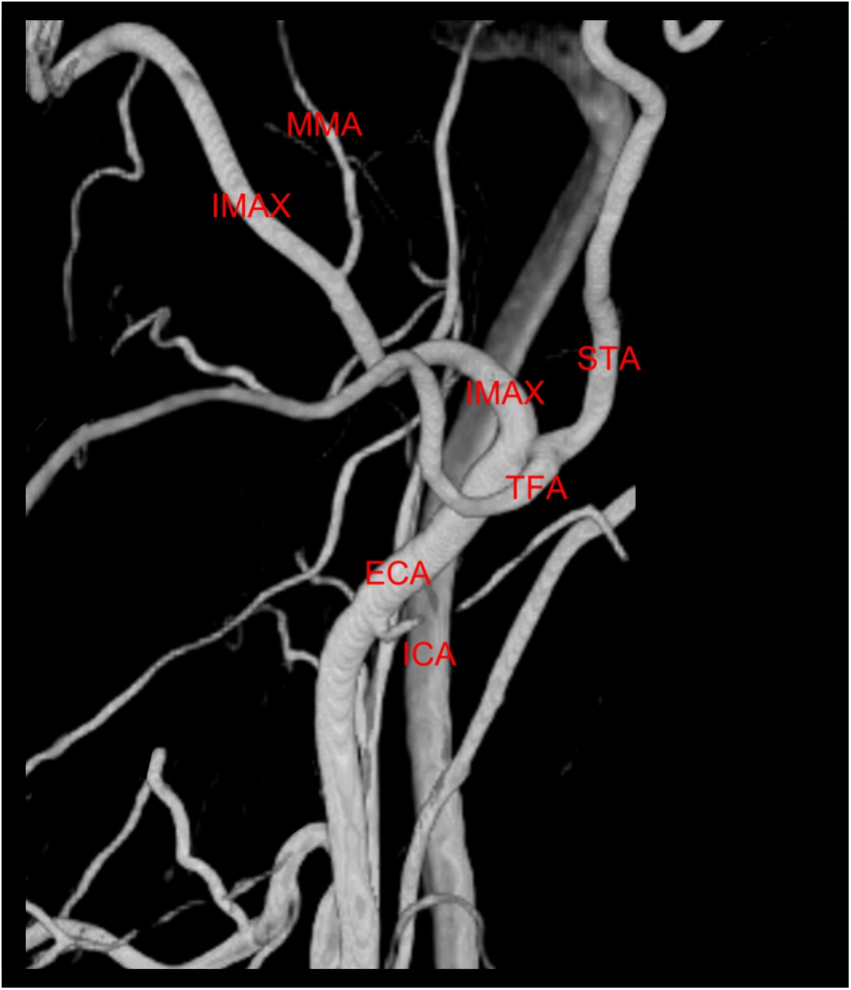
Lateral view of 3D digital subtraction angiography of the left external carotid artery demonstrates a normal appearance of the left internal maxillary artery (IMAX).

ECA, external carotid artery; ICA, internal carotid artery; MMA, middle meningeal artery; STA, superficial temporal artery; TFA, transverse facial artery.

During the angiographic runs of the right ECA, there was an incidental finding of a duplicated IMAX positioned medially to the lateral pterygoid muscle (Figures 1 to 4). The duplication was found to be 9 mm distal to the bifurcation of the ECA into the superficial temporal artery (STA) and IMAX at its transition from the first to the second maxillary segment with the middle meningeal artery (MMA) arising from the dominant superior segment of the duplication. The accessory meningeal artery arose proximal to the duplication as a common trunk with the inferior dental (alveolar) artery. Each duplication segment measured 1 cm in length before coalescing to a single lumen along the more distal course of the IMAX, that is, the transition from the second to the third IMAX segment.

## Discussion

Embryologically, the maxillary artery originates as a vascular network within the pterygoid mass of myoblasts, forming a ring surrounding the developing mandibular nerve with a superficial and deep component.^5^ Normally there is a regression of one half of the ring, resulting in either a superficial or deep course of the second part of the maxillary artery relative to the lateral pterygoid muscle. However, in the present case, there was the persistence of both halves of the ring, resulting in a duplicated right maxillary artery medial to the lateral pterygoid muscle and with separate origins from the MMA and accessory meningeal arteries.

A superficial course of the maxillary artery is reported in cadaveric studies of Japanese adults in 89.9% to 96.4% of the time^6^ and is associated with a common trunk of the MMA and accessory meningeal arteries. In contrast, with a deep course of the maxillary artery, there is a separate origin of the MMA and accessory meningeal arteries.^7^ In addition, a series of studies on Caucasian populations revealed a superficial course in approximately 61.6%.^6^

From an embryological perspective, the hyoid artery forms the stapedial artery, which further develops and branches into maxillomandibular and supraorbital branches. The ventral pharyngeal artery merges with the maxillomandibular division to form the ECA and the maxillary artery.^7^ The dorsal branch of the supraorbital division then develops into the MMA.

Aland and Shaw described a cadaveric case of a divided maxillary artery with two separate superior and inferior branches, with the inferior branch piercing the lower head of the lateral pterygoid muscle.^8^ The superior branch gave off the anterior and posterior deep temporal arteries, and the inferior branch gave off the infraorbital artery. Tadokoro et al. reported a cadaveric dissection with a similarly divided maxillary artery with deep and superficial branches.^9^ Claire et al. described a ring-like arrangement of the IMAX with a bifurcation into a major branch superficial to the lateral pterygoid muscle and a smaller deep branch that rejoined to form a loop.^5^ Maeda et al. also reported a case of a divided and reunited maxillary artery.^6^ More recently, and using CT imaging, Rusu et al. described an arterial “fenestration” of the maxillary artery with superior and inferior arms.^10^

Given the abovementioned embryology, the described anatomic variant is better denominated as a duplication. It is not a fenestration, given that another anatomical structure (e.g. nerve, vessel) does not run through the IMAX at the duplication site. Fenestrations are also usually found in the internal carotid or vertebral artery. Similarly, the duplicated IMAX does not represent segmentally unfused arteries (a non-fusion event) since one portion of the embryological superficial or deep ring regresses rather than fuses during development. Unfused segments are more commonly encountered in the basilar artery resulting from incomplete midline fusion of the paired ventral longitudinal arteries.^11^

Although rare in the IMAX, duplications have been more commonly described in the ICA, anterior cerebral artery (ACA), and at the vertebrobasilar junction. ICA duplications occur due to the recruitment of arteries from the ascending pharyngeal artery system in the congenital absence of one or several ICA segments.^12^ ACA duplications occur in up to 4% of the population^13^ and arise from duplicated vessels and the persistence of the ACA's infraorbital origin. Vertebrobasilar junction duplications can occur in the presence of a persistent primitive lateral vertebrobasilar anastomosis.^14^

In the case of our patient, who presented with an acute oropharyngeal hemorrhage, the atypical appearance of the IMAX raised the possibility of arterial dissection, given that the hemorrhage appeared to have originated near the site of the duplication. However, there was no positive radiological evidence of dissection with a lack of a flap, false lumen, or evidence of an intramural hematoma. Arterial dissection is also most common in the extracranial carotid artery. It occurs following blunt trauma to the neck or in connective tissue disorders, neither of which was the case in our patient.

In conclusion, we report a rare case of a duplicated IMAX for the first time using 3D digital subtraction angiography. This variation has previously only been described in cadaveric and CT studies. While this is an exceedingly rare variant, understanding the embryology and development of the IMAX will aid in identifying this variation when encountered in clinical practice. This is especially relevant when considering embolizing branches of the IMAX for any clinical indication.

## References

[1] Wollschlaeger G Wollschlaeger PB Lucas FV , et al. Experience and result with postmortem cerebral angiography performed as routine procedure of the autopsy. Am J Roentgenol Radium Ther Nucl Med 1967; 101: 68–87. 10.2214/ajr.101.1.68 6037344

[2] Lasjaunias P Berenstein A ter Brugge K . Surgical neuroangiography: clinical vascular anatomy and variations. Berlin, Brandenburg, Germany: Springer, 2001, pp. 479–629.

[3] Osborn RE Kirk G . Cerebral arterial fenestration. Comput Radiol 1987; 11: 141–145. 3608460 10.1016/0730-4862(87)90039-4

[4] Islak C Kocer N Kantarci F , et al. Endovascular management of basilar artery aneurysms associated with fenestrations. AJNR Am J Neuroradiol 2002; 23: 958–964. PMID: 12063224; PMCID: PMC7976902. 12063224 PMC7976902

[5] Claire PG Gibbs K Hwang SH , et al. Divided and reunited maxillary artery: developmental and clinical considerations. Anat Sci Int 2011; 86: 232–236. 21503610 10.1007/s12565-011-0106-x

[6] Maeda S Aizawa Y Kumaki K , et al. Variations in the course of the maxillary artery in Japanese adults. Anat Sci Int 2012; 87: 187–194. 23011579 10.1007/s12565-012-0146-x PMC3505518

[7] Tanoue S Kiyosue H Mori H , et al. Maxillary artery: functional and imaging anatomy for safe and effective transcatheter treatment. Radiographics 2013; 33: e209–e224. 10.1148/rg.337125173 24224604

[8] Aland RC Shaw V . Divided maxillary artery in relation to the lateral pterygoid muscle. Anat Sci Int 2016; 91: 207–210. 26077959 10.1007/s12565-015-0289-7

[9] Tadokoro O Umemura Y Utsuno H , et al. A case of a divided maxillary artery in the infratemporal fossa. Okajimas Folia Anat Jpn 2008; 85: 97–101. 19227200 10.2535/ofaj.85.97

[10] Rusu MC Vrapciu AD Popescu ŞA . Fenestrated Maxillary Artery. J Craniofac Surg 2022; 33: e861–e863. DOI: 10.1097/SCS.0000000000008788. 35882049

[11] Krings T Baccin CE Alvarez H , et al. Segmental unfused basilar artery with kissing aneurysms: report of three cases and literature review. Acta Neurochir (Wien) 2007; 149: 567–574. 17514352 10.1007/s00701-007-1118-0

[12] Krings T Lasjaunias PL . Segmental agenesis of the internal carotid artery distal to the posterior communicating artery leading to the definition of a new embryologic segment. AJNR Am J Neuroradiol 2006; 27: 246–247. PMID: 16484382; PMCID: PMC8148757. PMC8148757 16484382

[13] Perlmutter D Rhoton AL Jr . Microsurgical anatomy of the anterior cerebral-anterior communicating-recurrent artery complex. J Neurosurg 1976; 45: 259–272. 948013 10.3171/jns.1976.45.3.0259

[14] De Caro R Parenti A Munari PF . Persistent primitive lateral vertebrobasilar anastomosis. Acta Neurochir (Wien) 1996; 138: 592–594. PMID: 8800337 8800337 10.1007/BF01411182

